# Tuberculosis of the Mouth

**Published:** 1916-09

**Authors:** Hamilton Stillson

**Affiliations:** Seattle, Wash.


### TUBERCULOSIS OF THE MOUTH.

BY HAMILTON STILLSON, M.D., PH.D., F.A.C.S., SEATTLE, WASH.

Even since Trudeau, of blessed memory, worked up the tuberculin reaction for the diagnosis of tuberculosis, we have found tuberculosis of the mouth much more common than we had formerly supposed. And, though practically all the authors hold that tuberculosis of the mouth is secondary to systemic infection, my own opinion is that systemic invasion usually takes place by way of the mouth or the respiratory tract.

As far back as 1904, I read a paper before the Washington State Medical Society on "The Role Played by the Nose and the Mouth in the Etiology of Tuberculosis," in which paper I used these words: "I believe that the possibility of infection through the gums and decayed teeth is not given due consideration. The gums in children are nearly always ragged and sore, and infants are permitted to bite on objects picked up from the floor, to such dirty rubber nipples, etc. Decayed teeth form ideal pockets for these germs. And, as Mr. Norman Bennett and Mr. Herbert Tilly have pointed out, the pulp of the teeth readily permits, and indeed invites extension of the invasion. Miller, of Berlin, mentions thirty-four different local or general diseases traced to the action of bacteria in the mouth, a prominent place in the catalogue being given to tuberculosis."

Recently, Robert Levy, of Denver, has issued a book devoted entirely to "Tuberculosis of the Mouth." A perusal of it will convince anyone of my early contention.

### POINTS OF CONTENTION.

The principal points of election are:

1. At the angle of the mouth (lips) where rhagades are common.

2. In the fissures of the gums and in decayed teeth.

3. In the crypts of the tonsils, the most usual site.

4. On the hard palate, usually at the site of the incisor foramen. And on the soft palate, near the uvula.

5. On the back wall of the pharynx. We are beginning to look with as much suspicion on pharyngeal follicles as we usually do on ocular phlyctenules.

6. Very rarely on the tongue, where it usually starts as a small granule on the dorsum, or on the edge, near the tip.

### DIAGNOSIS.

The usual manifestations of tuberculosis of the mouth are:

1. The superficial apthous ulcer. This ulcer is pale, with non-inflammatory edges; it has a sort of worm-eaten appearance; is irregular in outline, except those in the early stage on the roof of the mouth; its surface is studded with yellowish spots and minute reddish elevations, and it bleeds very easily.

2. Infiltration presenting the appearance of granulation tissue.

3. The polypoidal tumor.

4. The deep ulcer, more often found in the aged.

5. The nodular lupus infiltrate, more often found on the face near the mouth, but sometimes found in the mouth.

The tumors mentioned are bosselated with tubercles, are soft and brittle, and bleed easily. The ulcers are dusted over with small red granulation, interspersed with yellow pin-head dots. There are usually very few tubercle bacilli to be found, but search should be made for them in the scrapings from the edge of the ulcers.

### DIFFERENTIAL DIAGNOSIS.

About the only other disease closely resembling tuberculosis of the mouth is syphilis. But:

1. In tuberculosis the mucous membrane is pale and anemic, while in syphilis it is somewhat cyanotic or copper-colored.

2. In tuberculosis the pain is never severe in the early stage, but in the latter stages it is much more so than it is in syphilis.

3. In tuberculosis the ulcer is seldom primary; in syphilis it is often primary on the lips and in the mouth.

4. In tuberculosis the ulcer is not punched out, as it is in syphilis; the edges are not undermined, and the margins are less elevated and less congested than they are in syphilis.

5. The cervical glands are not noticeable enlarged in the early stages of tuberculosis, as they are in syphilis.

6. In tuberculosis there is often considerable edema of the soft palate and uvula, the mucous membrane being studded over with millet-seed yellowish vesicules that give it an appearance of sudamina of the skin; while in syphilis the pillars of the fauces are the more often involved, their mucous membrane being somewhat cyanotic, especially in the tertiary stage. In the secondary stage, the first appearance is in the so-called mucous patch. This is really not an ulcer, but simply a necrosis of the superficial epithelia.

7. But, of course, the microscope, inoculation tests, tuberculin injection tests, temperature curves, etc., may all be necessary to establish a diagnosis; and even then the case may be a mixed one, requiring medication to bring matters to a conclusion.

Fortunately, tuberculosis of the mouth is characterized by a torpid course and a distinct tendency to cicatrization. There seems to be something in the secretions of the nose and the mouth peculiarly inimical to the tubercule bacillis. I can not recall in all history a single case of tuberculosis of the salivary gland. But much will depend on the condition of the general system and the progress of the disease in the lungs. Of course diseased teeth, tonsils and adenoids should be removed, cleansing washes used assiduously, and careful regulation of habits and diet made. If granulations are exuberant, they should be rubbed with lactic acid; if the ulcers are too painful, a little orthoform dusted on them will quiet them temporarily. Where it is necessary to cauterize more deeply, the effluv from a high frequency electrode is excellent for that purpose; where stimulation of the ulcer is desired, a stream of hot air directed against the sore will be found sufficient. Tuberculin in very small and repeated doses often acts charmingly. Formerly, tuberculosis of the mouth, like tuberculosis of the eye, was given over to despair, but in recent years I personally have grown quite hopeful of curing it.—*Northwestern Journal of Dentistry.*

